# The weekend effect in aneurysmal subarachnoid haemorrhage: a single centre experience and review

**DOI:** 10.1007/s10143-023-01973-x

**Published:** 2023-03-24

**Authors:** Daniel Murray, Abdurehman Choudhry, Daniel Rawluk, John Thornton, Alan O’Hare, Sarah Power, Matthew Crockett, Stephen MacNally, Paula Corr, Deirdre Nolan, Deirdre Coffey, Paul Brennan, Mohsen Javadpour

**Affiliations:** 1 National Centre for Neurosurgery, Beaumont Hospital, Dublin, Ireland; 2 Royal College of Surgeons in Ireland, St. Stephen’s Green, Dublin, Ireland; 3 Department of Neuroradiology, Beaumont Hospital, Dublin, Ireland; 4 Trinity College Dublin, Dublin, Ireland

**Keywords:** Aneurysm, Subarachnoid haemorrhage, Weekend effect

## Abstract

Clinical outcomes for patients admitted to hospital during weekend hours have been reported to be poorer than for those admitted during the week. Aneurysmal subarachnoid haemorrhage (aSAH) is a devastating form of haemorrhagic stroke, with a mortality rate greater than 30%. A number of studies have reported higher mortality for patients with aSAH who are admitted during weekend hours. This study evaluates the effect of weekend admission on patients in our unit with aSAH in terms of time to treatment, treatment type, rebleeding rates, functional outcome, and mortality. We analysed a retrospective database of all patients admitted to our tertiary referral centre with aneurysmal subarachnoid haemorrhage between February 2016 and February 2020. Chi-square tests and *t*-tests were used to compare weekday and weekend demographic and clinical variables. Univariate and multivariate logistic regression analyses were performed to assess for any association between admission during weekend hours and increased neurological morbidity (assessed via Glasgow Outcome Scale at 3 months) and mortality. Of the 571 patients included in this study, 191 were admitted during on-call weekend hours. There were no significant differences found in time to treatment, type of treatment, rebleeding rates, neurological morbidity, or mortality rates between patients admitted during the week and those admitted during weekend hours. Weekend admission was not associated with worsened functional outcome or increased mortality in this cohort. These results suggest that provision of 7-day cover by vascular neurosurgeons and interventional neuroradiologists in high-volume centres could mitigate the weekend effect sometimes reported in the aSAH cohort.

## Introduction

Aneurysmal subarachnoid haemorrhage (aSAH) is a devastating cerebrovascular event. The estimated incidence is generally agreed to be in the order of 6–7 per 100,000 person years in most populations [1], although this may be decreasing (possibly due to reduced smoking rates) [2, 3]. Current best practice guidelines advise prompt diagnosis and securing of the ruptured aneurysm [2, 4, 5]. The objective of early intervention is to prevent rebleeding (which is often a fatal event), as well as to limit secondary damage to the brain as a consequence of the primary haemorrhage [6, 7]. Previous research across a range of different pathologies has demonstrated an association between weekend admission and unfavourable clinical outcomes, such as increased morbidity and mortality [8–12]. Though the precise causes for this phenomenon are unclear, theories offered to explain it include reduced staffing levels, the absence of expert senior physicians on site, and decreased availability of imaging during weekend hours [9, 11]. A number of studies investigating the effect of weekend admission on patients with aSAH have reported a significant effect, by way of delays in treatment [13], and increased risk of both in-hospital mortality and 3-month mortality [4, 14, 15]. This study aims to investigate the effect of weekend admission on functional outcome and mortality (both in-hospital and at 3 months) on patients presenting with aSAH, in an institution with 7-day consultant cover by both vascular neurosurgeons and interventional neuroradiologists.

## Methods

### Study population

We analysed a database of patients admitted to our institution, a tertiary referral centre, with aSAH between February 2016 and February 2020. Institutional approval was obtained for the recording and use of this data. Our catchment area was inclusive of a population of 3.7 million people, covering the large majority of the geographical area of the Republic of Ireland (45,598 km^2^). Between elective and emergency cases, the centre treats over 300 aneurysm patients per year.

As this is a tertiary referral centre, the majority of patients first attended their local hospital for initial investigations and were transferred to the neurosurgical centre after the diagnosis of aSAH was made. SAH was confirmed on CT scan or lumbar puncture, while presence of an offending aneurysm was confirmed on CT angiography (CTA) and/or digital subtraction angiography (DSA). Treatment initially consisted of admission to a high-dependency unit or ICU, administration of intravenous fluid therapy and nimodipine, and close neurological observations (to monitor for any clinical evidence of the development of secondary effects such as hydrocephalus or vasospasm—if vasospasm was diagnosed, it was typically treated by hypertensive therapy and/or balloon or chemical angioplasty). Aneurysm treatment was provided on a 7-day per week basis by a team of 5 interventional neuroradiologists and 3 neurovascular surgeons. Weekday admission was defined as being from 00:00 h on Monday to 15:59 h on Friday, while weekend admission was defined as being from 16:00 on Friday to 23:59 on Sunday (or Monday, on public holidays). On admission to our unit, data was collected on numerous variables including age, gender, smoking status, history of hypertension, baseline World Federation of Neurosurgical Societies (WFNS) grade, Fisher grade, aneurysm-securing intervention, presence of clinical vasospasm, rebleeding, CSF diversion procedures, time from diagnosis to admission to the neurosurgical centre and time from admission to the neurosurgical centre to treatment, length of stay, in-hospital and 90-day mortality, and functional outcome at 3 months, using the Glasgow Outcome Scale (GOS).

### Statistical analysis

Chi-square tests and *t*-tests were used to compare the demographic and clinical variables of the weekday and weekend groups, for categorical data and means respectively. The principal outcomes of interest were the differences in treatments delivered (endovascular therapy or surgical clipping; CSF diversion procedures), treatment times, functional outcome, and mortality between the weekday admission group and weekend admission group.

With regard to functional outcome and mortality, univariate analyses were performed to assess for any significant relationships. All variables with *p* < 0.2 were included in multivariate analysis. Binomial regression was performed to analyse the effect of multiple variables, including weekend admission, on mortality. Independent variables included age, gender, history of hypertension, smoking history, Fisher grade, WFNS grade, presence of clinical vasospasm, rebleed status, and weekend admission. A separate specific regression analysis was performed to assess the effect of weekend admission on functional outcome. GOS of ≤ 3 was deemed to indicate a poor functional outcome. Statistical significance was defined as a *p* value of < 0.05. The data was analysed using SPSS (IBM, Version 26).

## Results

### Demographics and clinical variables

Five hundred and seventy-one patients were admitted to the neurosurgical centre with radiologically confirmed aSAH during the study period. Of these patients, 92% attended their local hospital first and were then transferred to our tertiary neurosurgical centre following the diagnosis of aSAH. The remaining 8% attended the neurosurgical centre directly. The mean age of patients was 55 ± 12 years. The majority (67.9%) were female. One hundred and ninety-one patients (32%) were admitted during weekend hours. Nine patients (1.5%) had negative CT brain imaging; in these cases, the diagnosis of SAH was ascertained via lumbar puncture. More than 97% of all patients had their diagnosis confirmed with DSA; the remainder were too unwell to undergo DSA but had their diagnosis confirmed on CTA. More than 87% of ruptured aneurysms were found in the anterior circulation. Almost one quarter (24%) of patients presented with poor neurological grade (WFNS Grades IV–V). There were no statistically significant demographic, clinical, or radiological differences between the weekday admission and weekend admission groups (Table 1).

**Table 1:** Demographic and clinical variables of the total study population

All patients	Total admissions (*n* = 571)	Weekday admissions (*n* = 380)	Weekend admissions (*n* = 191)	*p* Value
Age (years, mean ± SD)	55.6 ± 12	55.3 ± 12	56.1 ± 12	0.45
Female	67.9% (388)	66.0% (251)	71.7% (137)	0.16
Smoker	63.0% (360)	63.6% (242)	61.7% (118)	0.65
Hypertension	35.9% (205)	34.4% (131)	38.7% (74)	0.31
WFNS grade				
I	48.5% (277)	48.7% (185)	48.2% (92)	0.91
II	22.4% (128)	21.9% (83)	23.6% (45)	0.64
III	4.4% (25)	4.2% (16)	4.7% (9)	0.78
IV	14.5% (83)	14.7% (56)	14.1% (27)	0.84
V	10.2% (58)	10.5% (40)	9.4% (18)	0.68
Fisher grade				
1	5.6% (32)	5.3% (20)	6.3% (12)	0.62
2	10.3% (59)	10.3% (39)	10.4% (20)	0.97
3	19.1% (109)	19.2% (73)	18.9% (36)	0.93
4	65% (371)	65.2% (248)	64.4% (123)	0.85
Mean scan-to-admission time	6.52 h	6.64 h	6.15 h	0.69
Mean scan-to-treatment time	1.69 days	1.79 days	1.48 days	0.25
Admission to treatment time	1.3 ± 2 days	1.3 ± 2 days	1.2 ± 2 days	0.70
WFNS I–III only	1.1 ± 1 day	1.07 ± 1 day	1.1 ± 1 day	0.78
Aneurysm location				
Anterior circulation	87.2% (498)	87.4% (332)	86.9% (166)	0.86
Posterior circulation	12.8% (73)	12.6% (48)	13.1% (25)	
Aneurysm treatment modality				
No treatment	5.6% (32)	4.5% (17)	7.9% (15)	0.09
Endovascular	81.6% (466)	81.0% (308)	82.7% (158)	0.62
Surgical	12.8% (73)	14.5% (55)	9.4% (18)	0.08
Vasospasm				
Not present	74.1% (423)	71.8% (273)	78.5% (150)	0.08
Present clinically	25.9% (148)	28.2% (107)	21.5% (41)	
CSF diversion procedures				
External ventricular drain	20.8% (119)	22.1% (84)	18.3% (35)	0.29
Lumbar drain	3.0% (17)	2.6% (10)	3.6% (7)	0.50
Length of stay				
Mean (days ± SD)	16.3 ± 15	16.9 ± 15	15.2 ± 16	0.21
Median	12 days	13 days	11 days	
Mode	10 days	8 days	10 days	
Pre-operative rebleed rate	5.78% (33)	5.79% (22)	5.76% (11)	0.98
In-hospital mortality	11.2% (64)	11.3% (43)	11.0% (21)	0.91
Mortality at 3/12	13.0% (74)	13.2% (50)	12.6% (24)	0.84
GOS ≤ 3 at 3/12	23.3% (133)	24.5% (93)	21.0% (40)	0.35

The majority of patients had their aneurysm treated by endovascular means: 81% of patients admitted during the week, and 82% of patients admitted over the weekend underwent an endovascular procedure (*p* = 0.62). Of the weekday group, 14.5% were treated with surgical clipping, compared to 9.4% of the weekend group (*p* = 0.08). A small number of patients with confirmed aSAH did not undergo any aneurysm-securing intervention, mainly due to clinical instability—4.5% (17) of the weekday group and 7.9% (15) of the weekend group (*p* = 0.09); none of these patients survived. With regard to clinical vasospasm, 28.2% of weekday admissions were diagnosed with the condition, compared to 21.5% of weekend admissions (*p* = 0.08). The pre-operative rebleed rate for weekday admissions was 5.79% (22 of 380), while for weekend admissions, it was 5.76% (11 of 191; *p* = 0.98). ‘Rebleeding’ was defined as a radiologically-confirmed increase of haemorrhage (subarachnoid, intraventricular, or intraparenchymal) on a repeat CT scan, following sudden clinical deterioration where diagnosis of aSAH had previously been established. There was no significant difference in mean length of stay between weekday admissions (16.9 ± 15 days) and weekend admissions (15.2 ± 16 days; *p* = 0.21). There was no significant difference in time between diagnostic scan and admission to the tertiary neurosurgical centre (mean of 6.64 h for weekday admission and 6.15 h for weekend admissions; *p* = 0.69) or time between diagnostic scan and definitive treatment (mean of 1.79 days during weekday hours and 1.48 days during weekend hours, *p* = 0.25) between the two groups. The difference in mean admission-to-treatment times for the whole group was not statistically significant (1.3 ± 3 days for weekday admission vs 1.2 ± 3 days for weekend admission; *p* = 0.70). The difference in mean admission-to-treatment time for the subgroup of ‘good grade’ (WFNS I-III) patients was not statistically significant (1.07 ± 1 day for weekday admission v 1.1 ± 1 day for weekend admission, *p* = 0.78).

It is of particular importance to note that the overall mean admission-to-treatment was significantly increased by a number of outlier cases where aneurysm-securing treatment was considerably delayed. This was typically due to poor clinical status at presentation and/or haemodynamic instability. We identified 21 cases where aneurysm-securing treatment was delayed for greater than 5 days. With these cases excluded, the mean time-to-treatment decreased to just 19 h, which is well within the recommended 24-h window. Six of the 21 delayed cases were admitted during weekend hours, while the remainder (15) were admitted during weekdays; there was no statistically significant difference found between rates of delayed treatment for weekend and weekday admissions (*p* = 0.68).

We also observed that many patients were treated the day after admission. For example, the majority of patients admitted on Sundays during the study period were in fact treated on Monday—out of 71 patients admitted on a Sunday, 27 were treated on Sunday, while 33 were treated on Monday (46%). It should be noted, however, that 43 of the 71 patients admitted on a Sunday were admitted after 5 pm. It should also be noted that the majority of patients admitted on Friday were treated on Saturday (44%), suggesting that for patients admitted after normal working hours, they were typically treated the next day (regardless of whether the day of admission was a weekday or weekend day).

There was no statistically significant difference found between the relative number of night-time procedures during week and weekend days—77% of coiling/clipping procedures on Saturdays and Sundays were performed between 9 am and 5 pm, while 75% of these procedures on Monday to Friday were performed between 9 am and 5 pm.

During weekend hours, 14 of 176 patients (7.95%) were treated between 7 pm and 7 am (our departmental ‘handover’ times, or possibly a more appropriate definition of ‘night-time’). During weekdays, 25 of 363 (6.89%) patients were treated after 7 pm or before 7 am (OR 1.1 (95% CI 0.59–2.30); *p* = 0.65). As such, there was no significant difference between the relative number of procedures performed at night-time during the study period.

### Weekend admission and functional outcome

Of 571 patients, 493 had their 3-month outcome recorded; this data was not recorded for 13.95% of weekday admissions and 13.61% of weekend admissions. The 3-month GOS was recorded during outpatient reviews, by standardised telephone call with the patient or carer/next of kin, or with the patient’s hospital if they remained an inpatient, by the Neurovascular nurse specialist. At a 3-month review, the rate of patients with a GOS of 2 or 3—that is, ‘functionally dependent’ (i.e., ‘minimally responsive’ or ‘conscious but dependent’)—in the weekday admission group was 10.7%, while it was 8.4% in the weekend group (*p* = 0.29). Conversely, the rate of ‘functionally independent’ patients (GOS of 4 or 5) at 3 months was 75.5% in the weekday group and 79% in the weekend group. Linear regression analysis was performed to assess the effect of weekend admission, age, gender, history of smoking, and history of hypertension, Fisher grade, WFNS grade, clinical vasospasm, and use of CSF diversion procedures on GOS at 3 months. This analysis revealed a strong association between WFNS grade and GOS at 3 months (*p* =  < 0.001), but no statistically significant relationship between day of admission and GOS at 3 months (*p* = 0.80).

### Weekend admission and mortality

The overall in-hospital mortality rate during the study period was 11.2% (64 patients). The weekday admission mortality rate was 11.3%, while the weekend admission mortality rate was 11.0% (*p* = 0.91). The overall in-hospital mortality rate of patients who had undergone definitive treatment via endovascular coiling or surgical clipping was 7.2% (39 patients). The in-hospital mortality rate for patients admitted during the week who underwent aneurysm-securing treatment was 8.2%, while the in-hospital mortality rate for such patients admitted during weekend hours was 5.1% (*p* = 0.21). At 3-month follow-up, the mortality rate for patients who had been admitted during the week was 13.2% and 12.6% for those admitted during weekend hours (*p* = 0.84). Logistic regression, performed to ascertain the effects of weekend admission, age, gender, history of smoking, history of hypertension, Fisher grade, WFNS grade, clinical vasospasm, and rebleeding on mortality, found no statistically significant relationship between weekend admission and mortality (*p* = 0.90). Thus, there were no statistically significant differences in in-hospital or 3-month mortality between patients admitted with aSAH during the week and those admitted during weekend on-call hours. The statistical model explained that 40% (Nagelkerke *R*^2^) of the variance in mortality and 89.8% of cases were correctly classified. Patients who suffered a pre-operative rebleed were found to have a nine-fold increase in overall mortality risk, which was independent of weekday or weekend admission status (*p* < 0.001). As would be expected, history of smoking (*p* = 0.035) as well as higher Fisher grade (*p* = 0.013) and WFNS grade (*p* < 0.001) were also found to be associated with increased mortality risk.

### Subgroup analysis: patients with WFNS IV–V

Of those patients presenting to our institution with poor neurological grade (WFNS grades IV–V), 96 were admitted during the week, while 45 were admitted over the weekend. The proportion of patients admitted with WFNS grade IV or V were comparable (25.2% weekday v 23.5% weekend, *p* = 0.65). No significant differences were found in demographic or clinical covariates between these two subgroups (Table 2). Half of all patients who had initially presented with poor neurological grade had a poor functional outcome at 3 months, characterised by a GOS ≤ 3 (50% weekday v 51% weekend, *p* = 0.90). The overall in-hospital mortality rate for poor grade patients was 29.8% (weekday 28.1% v weekend 33.3%; *p* = 0.53), while mortality at 3-month follow up was found to be 33.3% (weekday 31.2% v weekend 37.8%; *p* = 0.43). For patients presenting with poor neurological grade, there were no significant differences found in functional outcome or mortality between those admitted during the week and those admitted during weekend hours.

**Table 2:** Demographic and clinical variables of patients admitted with WFNS grades IV–V

WFNS grades IV–V only	Total (*n* = 141)	Weekday (*n* = 96)	Weekend (*n* = 45)	*p* Value
Admission to treatment time	1.5 ± 3 days	1.6 ± 3 days	1.0 ± 2 days	0.32
In-hospital mortality	29.8% (42)	28.1% (27)	33.3% (15)	0.53
Mortality at 3/12	33.3% (47)	31.2% (30)	37.8% (17)	0.43
GOS ≤ 3 at 3/12	50.3% (71)	50.0% (48)	51.1% (23)	0.90

## Discussion

The ‘weekend effect’ is a well-documented phenomenon in the literature and a recognised feature of numerous time-critical pathologies such as ruptured abdominal aortic aneurysm, pulmonary embolism, and ischaemic stroke [8, 11]. Our study of 571 patients with aSAH found no association between weekend admission and mortality or functional outcome.

Five separate large-scale studies investigating the weekend effect in aSAH in the USA have been performed using Nationwide Inpatient Sample (NIS) data [13–17]. The results from these papers have been conflicting, with three studies reporting increased mortality for weekend admissions and two more reporting no significant effect. Though benefitting from very large sample sizes, the studies utilising this database have acknowledged limitations in this context: They use coding (ICD-9) rather than clinical diagnosis which may result in errors; they only include data for Saturday and Sunday and thus miss other ‘on-call weekend’ time periods and do not account for statutory holidays; they lack important data on variables such as grade or severity of haemorrhage, which means that validated scoring systems (such as Fisher grade and WFNS grade) cannot be included in analysis; and they lack data on functional outcome [13–17].

The factors which have been reported to account for the weekend effect include the following: that there are often fewer staff on site and that those present have less seniority and experience; that weekend on-call staff may be covering other healthcare staff and be managing patients with whom they are not familiar; that there are less supervisors on weekends, who are supervising staff they may not be familiar with; that imaging may not be as readily available; and that there can be delays in performing invasive treatments [8, 9, 11]. We hypothesise that having on-call weekend consultant Interventional Neuroradiology and Vascular Neurosurgery cover for both endovascular and neurosurgical treatment of aSAH patients could negate many of the aforementioned issues, as timely treatment will be performed, and the optimum management plan will be defined and carried out regardless of day of admission.

Three smaller-scale studies similar to the present one have been published, from the USA, the UK and China. The Chinese study reported no weekend effect [18], while the US and UK studies reported increased mortality risk with weekend admission [4, 19]. The UK, which has a health system most comparable to our own, published its National Confidential Enquiry into Patient Outcome and Death (NCEPOD) report on subarachnoid haemorrhage management in 2013, which recommended that, at least for good-grade patients, aneurysm-securing procedures should take place within 48 h of ictus [20]. Data from neurosurgical units in the UK have described delays in treatment during weekend hours, especially for patients with poor neurological grade, both prior to and after the publication of the NCEPOD report [21–26]. A recent paper from Germany examining the weekend effect on outcome after surgical clipping of ruptured aneurysms reported that, in a centre with the availability of out-of-hours clipping as well as standardised treatment protocols in place, admission outside of regular working hours did not affect patient outcomes [27].

A separate notable finding in our analysis was that those patients in our study who suffered a rebleed prior to treatment had significantly worse functional outcomes and higher mortality, a finding which has been documented in previous literature on the subject. The majority of these patients deteriorated within 6 h of diagnosis. Additional analysis is certainly indicated to understand the risk factors for ‘ultra-early’ rebleed in aSAH, in order to develop a predictive model to identify these vulnerable patients as promptly as possible. In this study, there was no significant difference seen in rebleed rates between patients admitted during weekday and weekend hours, likely due to the fact that there was no corresponding difference in time to treatment between the two groups.

Weekend admission has previously been shown to be an independent risk factor for increased mortality in patients with aSAH, especially affecting those with poor neurological grade [4]. Though there is certainly heterogeneity in the published data about the phenomenon of the weekend effect in aSAH patients [16–18], much of the published literature on the topic has reported an effect, most often in terms of increased mortality.

Our study provides further important evidence in the ongoing discussion over the ‘weekend effect’ in aneurysmal subarachnoid haemorrhage. Our data suggests that there is no significant aSAH weekend effect in our institution, which may be explained by the system of 7-day on-call cover by both interventional neuroradiologists and vascular neurosurgeons, with early aneurysm treatment regardless of day of admission.

There are recognised limitations to this study: primarily that this is a retrospective study and that the data is gathered from a single institution, which does limit generalisability.

## Conclusion

We examined the effect of weekend admission in a cohort of 571 aSAH patients admitted to our institution over a 4-year period. Weekend admission was not found to be associated with increased mortality or worsened functional outcome. Our results suggest that provision of seven-day cover by vascular neurosurgeons and interventional neuroradiologists in high-volume centres could mitigate the weekend effect sometimes reported in aSAH cohorts.

## Data Availability

Datasets are available upon request via the corresponding author.
